# ORAL HYGIENE AND THE INCIDENCE OF FUNCTIONAL DECLINE IN OLDER ADULTS AGED ≥75 YEARS: THE OHSAKA STUDY

**DOI:** 10.1093/geroni/igae098.2491

**Published:** 2024-12-31

**Authors:** Naoko Otsuki, Tomoaki Mameno, Satoko Takeuchi, Kazunori Ikebe, Ayumi Kono, Ryohei Yamamoto

**Affiliations:** Osaka University, Suita, Osaka, Japan; Osaka University Graduate School of Dentistry, Suita, Osaka, Japan; Osaka University Graduate School of Dentistry, Suita, Osaka, Japan; Osaka University Graduate School of Dentistry, Suita, Osaka, Japan; Osaka Metropolitan University, Osaka, Osaka, Japan; Osaka University, Suita, Osaka, Japan

## Abstract

Poor oral health has been identified as a risk factor for overall health. Management of oral problems is highly significant in the field of public health. This retrospective cohort study aimed to clarify the association between the oral hygiene and the incidence of functional decline in 134,957 participants aged 75 years or older, who underwent public dental checkups in Japan. The oral hygiene levels were scored by dentists as excellent, good, or poor. The outcome was the identification of functional decline based on Long-term Care Insurance certification. An association between the oral hygiene and the incidence of functional decline were assessed using the Fine and Gray model adjusted for clinically relevant factors. During the median observational period of 36 months, the incidence of functional decline was observed in 3,360 (5.3%) men and 2,693 (3.8%) women. The multivariable-adjusted model showed that poor oral hygiene was associated with functional decline in both men and women, respectively (Sub-hazard ratios [95% confidence interval] of Excellent, Good, and Poor: 1.00 [reference], 1.10 [0.99–1.24], and 1.57 [1.33–1.84], respectively for men; 1.00 [reference], 1.29 [1.14–1.46], and 1.56 [1.23–1.97], respectively for women). The findings of the present study suggest that municipal healthcare planning and home care should recommend oral hygiene in older adults aged ≥75 years.

